# Association between C-reactive protein and sarcopenia: The National Health and Nutrition Examination Survey

**DOI:** 10.1097/MD.0000000000041052

**Published:** 2024-12-27

**Authors:** Yang Li, Zhi-Wen Zhang

**Affiliations:** a Department of Orthopedic Surgery, Affiliated Hospital of Hubei University of Chinese Medicine, Wuhan, Hubei, China; b Hubei Provincial Hospital of Traditional Chinese Medicine, Wuhan, Hubei, China; c Hubei Provincial Institute of Traditional Chinese Medicine, Wuhan, Hubei, China; d Hubei Key Laboratory of Theory and Application Research of Liver and Kidney in Traditional Chinese Medicine (Hubei Province Hospital of Traditional Chinese Medicine).

**Keywords:** C-reactive protein, cross-sectional study, nutrition surveys, sarcopenia

## Abstract

C-reactive protein (CRP), a clinical biomarker, is frequently used to evaluate the inflammatory status of the body. However, the association between CRP levels and sarcopenia among the general adult population in the USA is unknown. This study focused on assessing whether CRP levels were associated with sarcopenia. This cross-sectional study collected adult data of adults from the 2015 to 2018 National Health and Nutrition Examination Survey. Four-extremity total muscle mass was used to evaluate sarcopenia (appendicular lean mass). In addition, dual-energy X-ray absorptiometry was adopted to measure appendicular lean mass. The CRP levels were used to assess inflammation status. Odds ratios (OR) and 95% confidence intervals (95% CI) were determined using multivariable logistic regression to analyze the association between CRP levels and sarcopenia. A multivariable-adjusted restricted cubic spline model was built to plot OR curves at 4 knots. Among the 3710 participants involved in this study (average age, 39.4 [11.54] years; 1801 [48.5%] men), 352 (9.5%) displayed characteristics of sarcopenia, while 3358 (90.5%) did not. Compared with participates in the lowest quartile (Q1) of CRP level (Q1; ≤0.08 to ≤0.7), those in the highest quartile (Q4; ≤4.3 to ≤188.5) had an adjusted OR for sarcopenia of 2.74 (95% CI, 1.65–4.57; *P* < .001). Based on the multivariable restricted cubic spline model, CRP levels showed a nonlinear association with sarcopenia (*P* < .001). The adjusted OR of sarcopenia of 1.86 (95% CI, 1.37–2.51; *P* < .001) was determined by 2 piecewise regression models for those having the CRP level of 1.8. Based on subgroup analysis, CRP levels were related to sarcopenia in males (OR, 1.03; 95% CI, 1–1.05) and individuals aged <50 years (OR, 1.03; 95% CI, 1.01–1.05), drinking (OR, 1.02; 95% CI, 1–1.03), and body mass index ≥ 25 kg/m^2^ (OR, 1.02; 95% CI, 1–1.03). Our results indicated that CRP levels showed a nonlinear correlation with sarcopenia among adults in the US.

## 1. Introduction

Sarcopenia, a Greek phrase implying fresh poverty, was initially depicted in 1989 by Rosenberg. Sarcopenia is an aging-related loss of skeletal muscle mass and strength.^[1,2]^ It is related to multiple pathophysiological events, including mitochondrial dysfunction, denervation, and hormonal and inflammatory alterations that negatively affect health, such as decreased function, falls, mortality, and frailty due to reduced lean body mass.^[3]^ Approximately 10% of individuals over 65 years of age are affected by the condition, including up to 30% of men over 80 years.^[4–6]^ This phenomenon will be complicated by the increase in the >60-year-old population, which may reach approximately 2 billion by 2050.

Although the pathogenesis of sarcopenia remains unknown, inflammation may have a critical effect. Inflammation is also associated with sarcopenia.^[4–6]^ With old age, the body may experience a phenomenon known as “inflammatory aging,” which is characterized by a persistent low-level inflammatory response. In this condition, the body produces excessive amounts of pro-inflammatory cytokines such as interleukin-6 (IL-6) and tumor necrosis factor-alpha (TNF-α). The pro-inflammatory cytokines are thought to promote muscle breakdown and inhibit muscle protein synthesis, which accelerates the loss of muscle mass. However, findings from previous studies on the relationship between C-reactive protein (CRP) levels and sarcopenia are limited.

CRP has been increasingly suggested as an acute inflammatory protein with a critical effect on infection responses and inflammatory processes in the host, such as apoptosis, the complement pathway, phagocytosis, nitric oxide production, and cytokine generation, especially TNF-α and IL-6.^[7–11]^ CRP activates many types of immune cells, including macrophages and dendritic cells, making them more active and involved in the inflammatory process, which helps the body to respond quickly to potential threats. CRP can also lead to an excessive inflammatory response. Understanding how CRP specifically affects these processes is important for the development of novel therapeutic approaches to aging-related diseases. However, the association between CRP level and sarcopenia needs to be further investigated.

To bridge the existing knowledge gap, the association of CRP with sarcopenia was assessed based on the National Health and Nutrition Examination Survey (NHANES) data. Therefore, we investigated whether CRP level was independently related to sarcopenia in adults from the 2015 to 2018 NHANES.

## 2. Materials and methods

### 2.1. Data source

The NHANES database is a cross-sectional, population-based survey that collects information on the health and nutrition of US household populations.^[5]^ In the current cross-sectional study, data were extracted from adult participants of the 2015 to 2018 NHANES cycles. These cycles were chosen because sarcopenia was only obtained from the Miscellaneous Pain Questionnaire. Data were collected between October 2023 and November 2023. There were 19,225 participants in the 2015 to 2018 NHANES cycles. This study excluded participants if the status of these participants was any of the following: (1) Their diet data were unavailable (n = 4185); (2) The data about sarcopenia were missing (n = 8009); (3) The data about covariates were missing (n = 3321). Finally, 3710 participants were included, of which 3358 participants without sarcopenia and 352 participants with sarcopenia.

### 2.2. Standard protocol approval, registration, and patient consent

In December 1998, the NHANES was approved by the NCHS Ethics Review Committee. Informed consent was obtained from all participants before enrollment. No additional Institutional Review Board approval was required to perform secondary analysis.

### 2.3. Study design and population

Participants aged over 20 years who completed the survey interviews were included in this study. Those with insufficient information regarding sarcopenia or CRP levels, those in pregnancy, and those without data for calculating CRP levels and covariates were eliminated. The exclusion criteria included those with insufficient information concerning sarcopenia or CRP, those who were pregnant, and those with missing data needed to calculate CRP and covariates.

### 2.4. Sarcopenia and CRP assessment

We evaluated sarcopenia using the total 4-extremity muscle mass (appendicular lean mass) with dual-energy X-ray absorptiometry using NHANES.^[12]^ Sarcopenia was defined as follows: The sarcopenia index was employed to define sarcopenia, with values of <0.789 and 0.512 indicating sarcopenia in male and female, respectively. Baseline CRP levels were determined and used as a continuous variable. The eventual outcome variable (dichotomous variable) was obtained based on current studies and guidelines.

The covariates included age, sex, race, marital status, education, drinking, smoking, hypertension, diabetes, body mass index (BMI), and CRP levels. The race was divided into Mexican American, other Hispanics, non-Hispanic White, non-Hispanic Black, and other race-including multiracial. Marital status was classified as married, widowed, divorced, separated, never-married, or living with a partner. Education level was classified as <9 years, 9 to 12 years, high school graduate, some college or AA degree, and college graduate or above. In line with the predetermined literature definitions, drinking status was classified as never drinking or drinking.

Underlying diseases (diabetes and hypertension) were defined in accordance with the questionnaire inquiries regarding whether the physician knew the condition previously. BMI was determined using a standardized approach based on height and weight. Latex-enhanced nephelometry was used to determine the CRP levels.

### 2.5. Statistical analysis

All estimates were calculated by accounting for NHANES sample weights. Categorical variables are shown as proportions (%), while continuous variables are presented as means (standard deviation) or medians (interquartile range). To compare intergroup differences, chi-square tests (categorical variables), one-way analysis of variance (normal distribution), and Kruskal–Wallis tests (skewed distribution) were conducted. To examine the correlation between sarcopenia and CRP levels, odds ratios (ORs) and 95% confidence intervals (95% CIs) were calculated. In Model 1, sociodemographic features, including age, sex, race, marital status, education, and drinking status were adjusted. Sociodemographic features and previous diseases were adjusted for in model 2. In addition, age, sex, marital status, race, education, drinking, smoking, diabetes, hypertension, and body mass index (BMI) were fully adjusted in Model 3.

The smoothing 2-piecewise logistic regression model was adopted to analyze the association threshold of sarcopenia with CRP in Model 3. In addition, inflection points were examined using the bootstrap resampling approach and the likelihood-ratio test.

We evaluated possible modifications for the association between sarcopenia and CRP, such as sex, age (<50 vs ≥50 years), drinking, and BMI (<25 vs =25 kg/m^2^). Multivariate logistic regression was used to evaluate inter-subgroup heterogeneity, while likelihood-ratio testing was performed to analyze subgroup-CRP interactions.

Free Statistics software version 1.9 and statistical software package R 3.3.2 (http://www.R-project.org) were used for statistical analysis. A 2-sided test was made for this descriptive research on every subject, and *P* < .05 represented statistical significance.

## 3. Results

### 3.1. Study population

**Figure 1.**
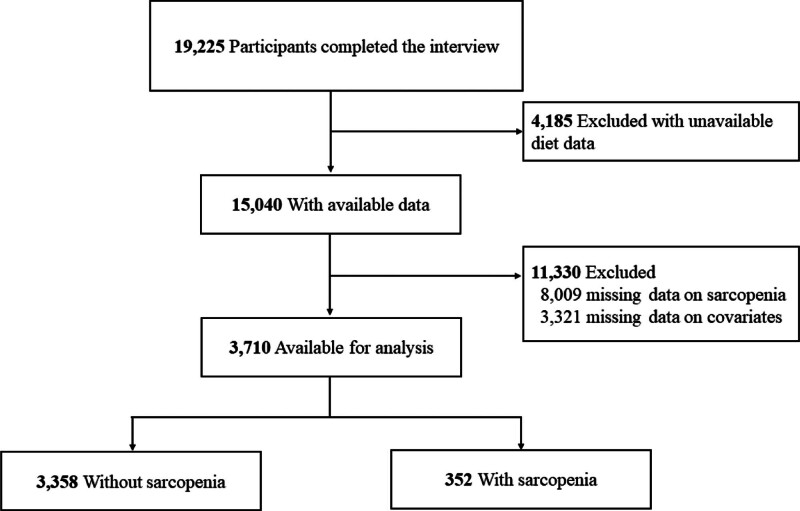
Inclusion and exclusion process was based on the 2015 to 2018 National Health and Nutrition Examination Survey.

A total of 19,225 participants completed the interview, including 15,040 participants with diet data. We excluded patients with no available information on sarcopenia (n = 8009) and covariates (n = 3321). Finally, 3710 participants were recruited from the 2015 to 2018 NHANES survey in the current cross-sectional study for analysis. Figure 1 presents more details regarding patient eligibility criteria.

### 3.2. Baseline features

Table 1 shows the baseline features of the participants based on the corresponding CRP quartiles. A total of 352 (9.5%) participants had sarcopenia. The mean age of all participants was (39.4 ± 11.5) years, including 1909 (51.5%) women. Those with higher CRP levels were usually older, female, married, non-Hispanic White, some college or AA degree, drinkers, a decreased risk of diabetes and hypertension, and obese.

**Table 1:** Characteristics of participants by quartiles of the CRP level in the NHANES 2015 to 2018 cycles.

Variables	Total (n = 3710)	Q1 (≤0.08 to ≤0.7)	Q2 (≤0.71 to ≤1.79)	Q3 (≤1.8 to ≤4.29)	Q4 (≤4.3 to ≤188.5)	*P*-value
Gender						<.001
Male	1801 (48.5)	514 (55.6)	498 (55.3)	468 (49.3)	321 (34.3)	
Female	1909 (51.5)	410 (44.4)	402 (44.7)	482 (50.7)	615 (65.7)	
Age	39.4 ± 11.5	36.8 ± 11.5	39.6 ± 11.3	40.5 ± 11.3	40.8 ± 11.3	<.001
Race						<.001
Mexican American	648 (17.5)	106 (11.5)	178 (19.8)	193 (20.3)	171 (18.3)	
Other Hispanic	414 (11.2)	87 (9.4)	97 (10.8)	112 (11.8)	118 (12.6)	
Non-Hispanic White	1231 (33.2)	298 (32.3)	292 (32.4)	309 (32.5)	332 (35.5)	
Non-Hispanic Black	737 (19.9)	185 (20)	153 (17)	192 (20.2)	207 (22.1)	
Other race-including multiracial	680 (18.3)	248 (26.8)	180 (20)	144 (15.2)	108 (11.5)	
Education level						<.001
<9	229 (6.2)	44 (4.8)	61 (6.8)	69 (7.3)	55 (5.9)	
9–12	374 (10.1)	74 (8)	89 (9.9)	101 (10.6)	110 (11.8)	
High school graduate	850 (22.9)	188 (20.3)	204 (22.7)	227 (23.9)	231 (24.7)	
Some college or AA degree	1255 (33.8)	301 (32.6)	289 (32.1)	328 (34.5)	337 (36)	
College graduate or above	1002 (27.0)	317 (34.3)	257 (28.6)	225 (23.7)	203 (21.7)	
Marital status						<.001
Married	1776 (47.9)	416 (45)	449 (49.9)	449 (47.3)	462 (49.4)	
Widowed	54 (1.5)	7 (0.8)	12 (1.3)	19 (2)	16 (1.7)	
Divorced	341 (9.2)	61 (6.6)	78 (8.7)	106 (11.2)	96 (10.3)	
Separated	131 (3.5)	24 (2.6)	30 (3.3)	34 (3.6)	43 (4.6)	
Never-married	925 (24.9)	282 (30.5)	232 (25.8)	214 (22.5)	197 (21)	
Living with partner	483 (13.0)	134 (14.5)	99 (11)	128 (13.5)	122 (13)	
Drinker						<.001
No	1074 (28.9)	246 (26.6)	233 (25.9)	273 (28.7)	322 (34.4)	
Yes	2636 (71.1)	678 (73.4)	667 (74.1)	677 (71.3)	614 (65.6)	
BMI	29.2 ± 6.9	24.5 ± 4.2	27.4 ± 4.9	30.8 ± 6.2	34.0 ± 7.7	<.001
Hypertension						<.001
No	2960 (79.8)	791 (85.6)	725 (80.6)	745 (78.4)	699 (74.7)	
Yes	750 (20.2)	133 (14.4)	175 (19.4)	205 (21.6)	237 (25.3)	
Diabetes						<.001
No	3294 (88.8)	880 (95.2)	837 (93)	824 (86.7)	753 (80.4)	
Yes	416 (11.2)	44 (4.8)	63 (7)	126 (13.3)	183 (19.6)	
Sarcopenia						<.001
No	3358 (90.5)	901 (97.5)	842 (93.6)	834 (87.8)	781 (83.4)	
Yes	352 (9.5)	23 (2.5)	58 (6.4)	116 (12.2)	155 (16.6)	

Mean + SD for continuous variables: *P* value was calculated by weighted linear regression model.

Percentage for categorical variables: *P* value was calculated by weighted chi-square test.

CRP = C-reactive protein, NHANES = National Health and Nutrition Examination Survey, SD = standard deviation.

### 3.3. Association between sarcopenia and CRP

As shown in Table 2, high CRP levels were correlated with a higher prevalence of sarcopenia when age, sex, marital status, race, educational level, BMI, hypertension, and diabetes were adjusted. Correlation was maintained if the CRP levels were converted to categorical variables as quartiles. Compared with participates with quartile1 (Q1) of CRP (≤0.08 to ≤0.7), those with Q3 (≤1.8 to ≤4.29) and Q4 (≤4.3 to ≤188.5) had the adjusted ORs for sarcopenia of 2.43 (95% CI; 1.49–3.98; *P* < .001) and 2.74 (95% CI; 1.65–4.57; *P* < .001).

**Table 2:** Association between CRP level and sarcopenia among adult participants in the NHANES 2015 to 2018 cycles.

	No	Crude OR (95% CI)	*P*-value	Model 1 OR (95% CI)	*P*-value	Model 2 OR (95% CI)	*P*-value	Model 3 OR (95% CI)	*P*-value
Q1	924	1 (Ref)		1 (Ref)		1 (Ref)		1 (Ref)	
Q2	900	2.7 (1.65–4.41)	<.001	2.17 (1.31–3.59)	.003	2.12 (1.28–3.51)	.003	1.6 (0.96–2.68)	.074
Q3	950	5.45 (3.45–8.61)	<.001	4.64 (2.9–7.42)	<.001	4.44 (2.77–7.11)	<.001	2.43 (1.49–3.98)	<.001
Q4	936	7.77 (4.97–12.17)	<.001	7.57 (4.75–12.08)	<.001	6.96 (4.35–11.14)	<.001	2.74 (1.65–4.57)	<.001
Trend test	3710	1.84 (1.65–2.06)	<.001	1.92 (1.69–2.17)	<.001	1.86 (1.64–2.11)	<.001	1.36 (1.18–1.57)	<.001

Model 1: Sociodemographic features, including age, gender, race, marital status, education, and drinking status were adjusted. Model 2: Sociodemographic features and previous disease were adjusted. Model 3: Age, gender, marital status, race, education, drinking, smoking, diabetes, hypertension, and body mass index (BMI) were fully adjusted.

CI = confidence interval, NHANES = National Health and Nutrition Examination Survey, OR = odds ratio, Ref = reference.

**Figure 2.**
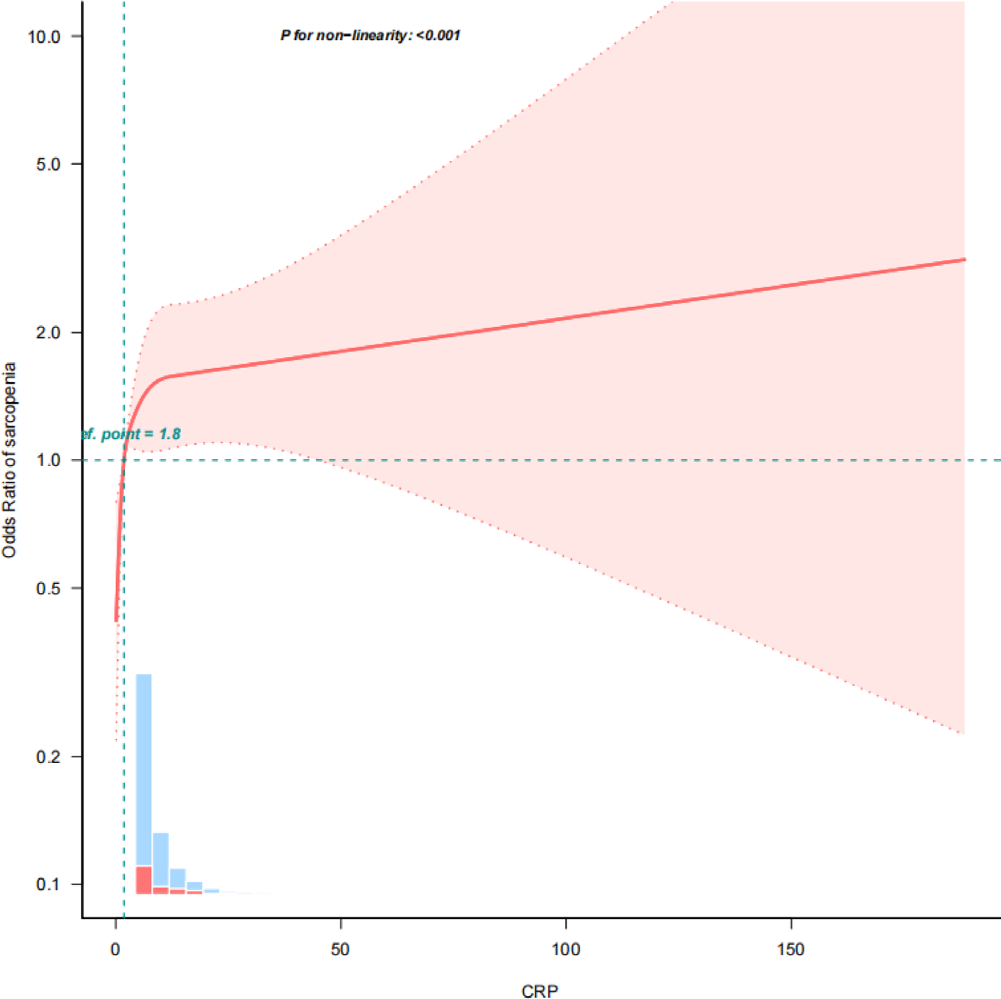
Association between CRP level and sarcopenia. Solid and dashed lines indicate the predicted value and 95% CI. CRP = C-reactive protein, CI = confidence interval.

Therefore, sarcopenia exhibited a nonlinear relation with CRP levels (*P* < .001) using the restricted cubic spline model (Fig. 2). Additionally, the adjusted OR of sarcopenia of 1.86 (95% CI, 1.37–2.51; *P* < .001) was obtained for participates with CRP levels ≥ 1.8, using 2-piecewise regression models (Table 3).

**Table 3:** Association between CRP level and sarcopenia using 2-piecewise regression models.

	Crude model		Adjusted model	
CRP level	OR (95%)	*P*-value	OR (95%)	*P*-value
<1.8	1 (Ref)		1 (Ref)	
≥1.8	3.61 (2.79–4.67)	<.001	1.86 (1.37–2.51)	<.001

Age, gender, marital status, race, education, drinking, smoking, diabetes, hypertension, and body mass index (BMI) were fully adjusted.

CRP = C-reactive protein, OR = odds ratio, Ref = reference.

### 3.4. Subgroup analysis

**Figure 3.**
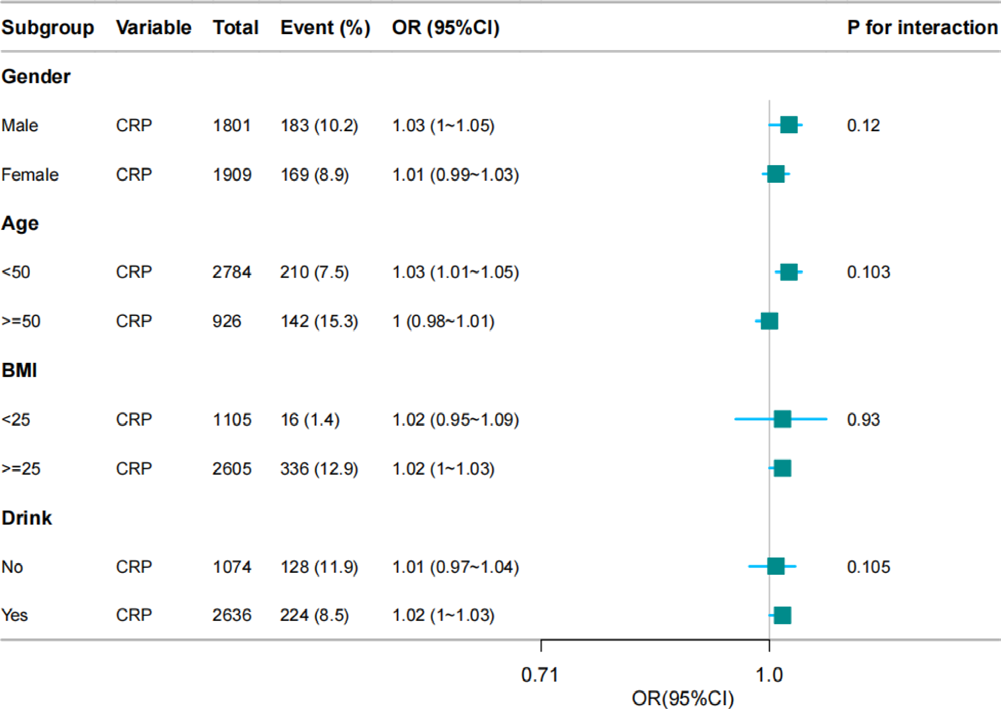
Association between CRP level and sarcopenia according to the general characteristics. CRP = C-reactive protein, OR = odds ratio, CI = confidence interval, BMI = body mass index.

The results of the subgroup analysis are shown in Figure 3. CRP levels were related to sarcopenia in males (OR, 1.03; 95% CI, 1–1.05) and individuals aged <50-year-old (OR, 1.03; 95% CI, 1.01–1.05), drinking (OR, 1.02; 95% CI, 1–1.03), and BMI ≥ 25 kg/m^2^ (OR, 1.02; 95% CI, 1–1.03). No association was found between CRP levels and sarcopenia in female or participants aged ≥50-years, those with no drinking, or those with BMI < 25 kg/m^2^ (Fig. 3).

## 4. Discussion

In the current nationwide cross-sectional study, CRP levels showed a nonlinear association with sarcopenia among the US adult population. The correlation was robust in the sensitivity and subgroup analyses.

Sarcopenia has been suggested to be closely related to inflammatory reactions. Chronic low-grade inflammation may occur during aging among older people.^[13]^ Skeletal muscle cells generate inflammatory cytokines during inflammation, causing sarcopenia, which is characterized by weak muscle strength, low muscle mass, and low physical performance, resulting in negative outcomes. Loss of mass, strength, and fitness of skeletal muscle within the 4 extremities in males and females are related to increased levels of pro-inflammatory cytokines, including IL-6, TNF-α, and CRP.^[14–16]^ However, many studies have been conducted on obese populations or those with additional chronic disorders.^[17–19]^ Therefore, their conclusions are likely to be influenced by these underlying diseases. As a result, the association between muscle mass and inflammatory cytokine levels remains unknown. In contrast to the previous results, we examined NHANES data by multivariable regression after adjusting for possible confounders. Thus, our findings could be generalized to all US adults. In this study, it was shown that the CRP levels were associated with sarcopenia in males (OR, 1.03; 95% CI, 1–1.05), individuals aged <50-year-old (OR, 1.03; 95% CI, 1.01–1.05), drinking (OR, 1.02; 95% CI, 1–1.03), and BMI ≥ 25 kg/m^2^ (OR, 1.02; 95% CI, 1–1.03). According to dose–response analyses, CRP levels showed a nonlinear relationship with sarcopenia. In particular, the risk of sarcopenia increased with increasing CRP levels in those with the CRP levels > 1.8. In addition, the association between CRP levels and sarcopenia was robust in the subgroup and sensitivity analyses.

In addition to contraction and relaxation, skeletal muscle is also a secretory organ.^[13,20,21]^ Recently, this view has been applied extensively. During skeletal muscle contraction, cytokines are generated and released. These cytokines are called myokines, which include IL-6, IL-8, and IL-15, and may influence the activity of skeletal muscle and metabolism of additional organs and tissues.^[22–24]^ Similar to typical hormones, myokines exert their effects through endocrine, autocrine, and paracrine mechanisms to change target tissue physiological activity. Interactions between skeletal muscle and additional organs or tissues are dependent on myokines.^[25–27]^ Exercises can upregulate the gene levels of certain cytokines in skeletal muscles and increase the levels of different circulating cytokines.^[28]^ However, cytokine gene levels in skeletal muscles may not be causally associated with increased circulating cytokines.^[16,29,30]^ These findings suggest that the physiological mechanism of resistance training is the present mainstream interventional measure to prevent sarcopenia. Physical activity and diet are key interventional measures for sarcopenia patients because they are important for modulating the systemic inflammatory state in patients with sarcopenia. Thus, investigating sarcopenia-related inflammatory cytokines is of great significance, as these cytokines are vital for diagnosing sarcopenia, developing innovative interventions, and evaluating their efficacy.

Dietary patterns have been suggested to be tightly related to sarcopenia.^[31,32]^ The association between dietary patterns and inflammation can be interpreted as a correlation with sarcopenia. In particular, the American diet results in increased CRP and IL-6 levels in the serum, while Dietary Approaches to Stop Hypertension and Mediterranean diets have decreased inflammatory factor levels. In a follow-up study, we investigated whether dietary patterns were associated with sarcopenia.

However, this study had several limitations. First, this study focused on the US population; thus, more investigations are needed to confirm whether our findings are generalizable to more populations. Second, the residual confounders were not eliminated. Multivariate logistic regression models were used in this study. Sensitivity and subgroup analyses were used to control for the impact of potential confounders on the association of CRP levels with sarcopenia. Third, due to the lack of sarcopenia occurrence or severity data in the NHANES database, we were unable to evaluate the correlation of CRP levels with the clinical characteristics of sarcopenia. Owing to its cross-sectional nature, it was impossible to determine the causal relationship between CRP levels and sarcopenia. Therefore, more longitudinal studies need to be performed to determine whether our observed correlation between CRP level and sarcopenia is causal.

## 5. Conclusions

Based on our results, CRP levels showed a nonlinear association with sarcopenia among adults in the United States. The CRP levels were related to sarcopenia in males, the individuals aged <50-year-old, drinking and BMI ≥ 25 kg/m^2^. In addition, the association between CRP levels and sarcopenia was robust in the subgroup and sensitivity analyses. Thus, it is necessary to consider the possible role of CRP levels in sarcopenia when treating and preventing these conditions. Therefore, the studies on the molecular mechanisms underlying the pathogenesis of sarcopenia should be performed to better prevent and treat sarcopenia.

## Acknowledgments

We appreciate all the staff members of our institution. We gratefully thank Dr Jie Liu from the Department of Vascular and Endovascular Surgery, Chinese PLA General Hospital, for his constant efforts in statistical support, consultations of study design, and comments on this study.

## Author contributions

**Data curation:** Yang Li.

**Funding acquisition:** Zhi-Wen Zhang.

**Methodology:** Yang Li.

**Supervision:** Zhi-Wen Zhang.

**Writing – original draft:** Yang Li.

## References

[1] Chen LK Liu LK Woo J. Sarcopenia in Asia: consensus report of the Asian working group for sarcopenia. J Am Med Dir Assoc. 2014;15:95–101. 24461239 10.1016/j.jamda.2013.11.025

[2] Cho MR Lee S Song SK. A review of sarcopenia pathophysiology, diagnosis, treatment and future direction. J Korean Med Sci. 2022;37:e146. 35535373 10.3346/jkms.2022.37.e146 PMC9091430

[3] Cruz-Jentoft AJ Bahat G Bauer J. Sarcopenia: revised European consensus on definition and diagnosis. Age Ageing. 2019;48:16–31. 30312372 10.1093/ageing/afy169 PMC6322506

[4] Hu Y Peng W Ren R Wang Y Wang G. Sarcopenia and mild cognitive impairment among elderly adults: the first longitudinal evidence from CHARLS. J Cachexia Sarcopenia Muscle. 2022;13:2944–52. 36058563 10.1002/jcsm.13081 PMC9745544

[5] Liu H Wang D Wu F Dong Z Yu S. Association between inflammatory potential of diet and self-reported severe headache or migraine: a cross-sectional study of the National Health and Nutrition Examination Survey. Nutrition. 2023;113:112098. 37356381 10.1016/j.nut.2023.112098

[6] Tournadre A Vial G Capel F Soubrier M Boirie Y. Sarcopenia. Joint Bone Spine. 2019;86:309–14. 30098424 10.1016/j.jbspin.2018.08.001

[7] Dix C Zeller J Stevens H. C-reactive protein, immunothrombosis and venous thromboembolism. Front Immunol. 2022;13:1002652. 36177015 10.3389/fimmu.2022.1002652 PMC9513482

[8] Osimo EF Baxter LJ Lewis G Jones PB Khandaker GM. Prevalence of low-grade inflammation in depression: a systematic review and meta-analysis of CRP levels. Psychol Med. 2019;49:1958–70. 31258105 10.1017/S0033291719001454 PMC6712955

[9] Pope JE Choy EH. C-reactive protein and implications in rheumatoid arthritis and associated comorbidities. Semin Arthritis Rheum. 2021;51:219–29. 33385862 10.1016/j.semarthrit.2020.11.005

[10] Sproston NR Ashworth JJ. Role of C-reactive protein at sites of inflammation and infection. Front Immunol. 2018;9:754. 29706967 10.3389/fimmu.2018.00754 PMC5908901

[11] Ullah I Awan HA Aamir A. Role and perspectives of inflammation and C-reactive protein (CRP) in psychosis: an economic and widespread tool for assessing the disease. Int J Mol Sci . 2021;22:13032. 34884840 10.3390/ijms222313032 PMC8657450

[12] Chen LK Woo J Assantachai P. Asian working group for sarcopenia: 2019 consensus update on sarcopenia diagnosis and treatment. J Am Med Dir Assoc. 2020;21:300–7.e2. 32033882 10.1016/j.jamda.2019.12.012

[13] Picca A Coelho-Junior HJ Calvani R Marzetti E Vetrano DL. Biomarkers shared by frailty and sarcopenia in older adults: a systematic review and meta-analysis. Ageing Res Rev. 2022;73:101530. 34839041 10.1016/j.arr.2021.101530

[14] Wu X Li X Xu M Zhang Z He L Li Y. Sarcopenia prevalence and associated factors among older Chinese population: findings from the China health and retirement longitudinal study. PLoS One. 2021;16:e0247617. 33661964 10.1371/journal.pone.0247617 PMC7932529

[15] Lin S Chen X Cheng Y. C-reactive protein level as a novel serum biomarker in sarcopenia. Mediators Inflamm. 2024;2024:3362336. 39502753 10.1155/2024/3362336 PMC11535261

[16] Shokri-Mashhadi N Moradi S Heidari Z Saadat S. Association of circulating C-reactive protein and high-sensitivity C-reactive protein with components of sarcopenia: a systematic review and meta-analysis of observational studies. Exp Gerontol. 2021;150:111330. 33848566 10.1016/j.exger.2021.111330

[17] Antuña E Cachán-Vega C Bermejo-Millo JC. Inflammaging: implications in sarcopenia. Int J Mol Sci . 2022;23:15039. 36499366 10.3390/ijms232315039 PMC9740553

[18] Kalinkovich A Livshits G. Sarcopenic obesity or obese sarcopenia: a cross talk between age-associated adipose tissue and skeletal muscle inflammation as a main mechanism of the pathogenesis. Ageing Res Rev. 2017;35:200–21. 27702700 10.1016/j.arr.2016.09.008

[19] Nardone OM De Sire R Petito V. Inflammatory bowel diseases and sarcopenia: the role of inflammation and gut microbiota in the development of muscle failure. Front Immunol. 2021;12:694217. 34326845 10.3389/fimmu.2021.694217 PMC8313891

[20] Purnamasari D Tetrasiwi EN Kartiko GJ Astrella C Husam K Laksmi PW. Sarcopenia and chronic complications of type 2 diabetes mellitus. Rev Diabet Stud. 2022;18:157–65. 36309772 10.1900/RDS.2022.18.157 PMC9652710

[21] Sanz-Cánovas J López-Sampalo A Cobos-Palacios L. Management of type 2 diabetes mellitus in elderly patients with frailty and/or sarcopenia. Int J Environ Res Public Health. 2022;19:8677. 35886528 10.3390/ijerph19148677 PMC9318510

[22] Cruzat V Macedo Rogero M Noel Keane K Curi R Newsholme P. Glutamine: metabolism and immune function, supplementation and clinical translation. Nutrients. 2018;10:1564. 30360490 10.3390/nu10111564 PMC6266414

[23] Kirk B Feehan J Lombardi G Duque G. Muscle, bone, and fat crosstalk: the biological role of myokines, osteokines, and adipokines. Curr Osteoporos Rep. 2020;18:388–400. 32529456 10.1007/s11914-020-00599-y

[24] Li G Zhang L Wang D. Muscle–bone crosstalk and potential therapies for sarco-osteoporosis. J Cell Biochem. 2019;120:14262–73. 31106446 10.1002/jcb.28946 PMC7331460

[25] Barbalho SM Flato UAP Tofano RJ. Physical exercise and myokines: relationships with sarcopenia and cardiovascular complications. Int J Mol Sci . 2020;21:3607. 32443765 10.3390/ijms21103607 PMC7279354

[26] Dumont NA Bentzinger CF Sincennes MC Rudnicki MA. Satellite cells and skeletal muscle regeneration. Compr Physiol. 2015;5:1027–59. 26140708 10.1002/cphy.c140068

[27] Tidball JG. Mechanisms of muscle injury, repair, and regeneration. Compr Physiol. 2011;1:2029–62. 23733696 10.1002/cphy.c100092

[28] Xie H Ruan G Wei L. The inflammatory burden index is a superior systemic inflammation biomarker for the prognosis of non-small cell lung cancer. J Cachexia Sarcopenia Muscle. 2023;14:869–78. 36852672 10.1002/jcsm.13199 PMC10067487

[29] Mesinovic J Zengin A De Courten B Ebeling PR Scott D. Sarcopenia and type 2 diabetes mellitus: a bidirectional relationship. Diabetes Metab Syndr Obes. 2019;12:1057–72. 31372016 10.2147/DMSO.S186600 PMC6630094

[30] Peixoto da Silva S Santos JMO Costa ESMP Gil da Costa RM Medeiros R. Cancer cachexia and its pathophysiology: links with sarcopenia, anorexia and asthenia. J Cachexia Sarcopenia Muscle. 2020;11:619–35. 32142217 10.1002/jcsm.12528 PMC7296264

[31] Baumgartner RN. Body composition in healthy aging. Ann N Y Acad Sci. 2000;904:437–48. 10865787 10.1111/j.1749-6632.2000.tb06498.x

[32] Vicente de Sousa O Mendes J Amaral TF. Association between nutritional and functional status indicators with caregivers’ burden in Alzheimer’s disease. Nutr Diet. 2022;79:380–9. 34031961 10.1111/1747-0080.12679

